# Semaglutide Induces Oxidative Stress and Differentially Modulates mTOR-Dependent Growth and Invasion in Human Trophoblast Cell Models: Implications for Placental Function

**DOI:** 10.3390/cimb48050524

**Published:** 2026-05-18

**Authors:** Elizabeth Thurmond, Eliza J. Roeth, Kristen Noyes, Madeline Boyer, Ethan Evans, Benjamin T. Bikman, Paul R. Reynolds, Juan A. Arroyo

**Affiliations:** 1 Department of Cell Biology and Physiology, Brigham Young University, 3052 LSB, Provo, UT 84602, USA; 2 School of Medicine, Brigham Young University, Provo, UT 84602, USA

**Keywords:** semaglutide, trophoblast, mTOR, invasion, ROS, mitochondria

## Abstract

Semaglutide, a long-acting glucagon-like peptide-1 receptor agonist (GLP-1RA), has transformed obesity and diabetes management. However, its expanding use among reproductive-age women raises concerns about potential effects on early placental development. We examined semaglutide’s impact on two human trophoblast cell lines: Swan71 (invasive extravillous) and BeWO (syncytiotrophoblast-like). Cells were treated with semaglutide (100 nM) for 24 h, and proliferation, viability, mitochondrial respiration, oxidative stress, signaling pathways, and invasiveness were evaluated. Semaglutide significantly reduced proliferation in Swan71 cells and increased it in BeWO cells, with no significant change in viability for Swan71 and a slight increase for BeWO. Western blot analysis revealed altered phosphorylation of key signaling proteins, including mTOR, p70S6K, 4EBP1, AKT, and ERK, as well as increased AMPK phosphorylation, indicating a shift toward catabolic signaling. Reactive oxygen species (ROS) accumulation increased markedly, accompanied by altered oxygen consumption rates—reduced in Swan71 cells and elevated in BeWO cells. Functionally, semaglutide suppressed Swan71 invasion through Matrigel by approximately three-fold. These findings suggest that semaglutide induces oxidative and metabolic stress in trophoblasts and is associated with altered mTOR-mediated signaling and reduced invasive potential. Such cellular alterations may contribute to compromised placental development and uterine vascular remodeling if exposure occurs near conception. While clinical data remain limited, this study provides mechanistic insight supporting caution in the use of semaglutide during the periconception period and underscores the need for targeted reproductive safety studies.

## 1. Introduction

In 2022, more than 1 billion adults were classified as obese globally—triple the 1975 rate—and projections suggest nearly 1.9 billion adults (approximately 25% of the global population) will be affected by 2035, with some estimates warning that over half of all adults worldwide could be overweight or obese by 2050 [1,2]. This escalating prevalence is driven by rapid urbanization, sedentary lifestyles, and widespread availability of calorie-dense, nutrient-poor foods [3].

Obesity extends beyond excess adiposity to promote development of chronic conditions such as type 2 diabetes, cardiovascular disease, and several cancers—non-communicable diseases that together account for more than 5 million deaths globally each year [2,4].

Pregnancy magnifies the physiological stresses of obesity, compounding health risks for both mother and baby. In the United States, nearly half of all pregnant women are considered obese, and similar patterns are emerging worldwide [4]. These women face a greater risk of complications such as gestational diabetes mellitus (GDM) compared to those of normal weight [4]. There’s also a higher incidence of hypertensive disorders like gestational hypertension and preeclampsia [4,5].

Obstetric complications are more frequent in obese pregnancies. Obese women are more likely to require cesarean sections, induced labor, and experience postpartum hemorrhage [6]. After delivery, these women face greater risks of blood clots, wound infections, and difficulties initiating breastfeeding [5,7]. For their babies, risks include premature birth, macrosomia, congenital abnormalities, stillbirth, and neonatal death [8,9]. In the long term, children born to obese mothers are more likely to develop metabolic disorders, including childhood obesity and insulin resistance—continuing the cycle across generations [8,10]. Despite the scale of the problem, many existing guidelines fall short in offering personalized strategies to manage weight in women who are pregnant or planning to conceive. These patterns highlight how maternal metabolism profoundly shapes pregnancy outcomes and underscores the need for tailored interventions.

While maternal obesity increases risks of gestational diabetes, hypertensive disorders, and macrosomia, severe or unintended weight loss during pregnancy is generally associated with higher risks of complications such as preterm birth, low birth weight, and adverse perinatal outcomes. This is particularly relevant for conditions involving significant caloric restriction (e.g., hyperemesis gravidarum or eating disorders). However, in women who begin pregnancy with obesity, modest weight loss or minimal weight gain may not always lead to adverse outcomes and can sometimes reduce risks of macrosomia or cesarean delivery. Given that semaglutide induces substantial weight loss, its potential direct and indirect (weight loss-mediated) effects on early placental development warrant careful mechanistic investigation.

Recently emerging therapeutics, namely glucagon-like peptide-1 receptor agonists (GLP-1RAs), have proven effective for obesity management, offering a new option to address these challenges. Semaglutide has demonstrated high efficacy. As a long-acting GLP-1 analog with 94% similarity to natural GLP-1, semaglutide works by stimulating receptors in the pancreas, gut, and brain [11]. This action enhances insulin release, lowers glucagon levels, slows down gastric emptying, and promotes feelings of fullness, all of which contribute to lower calorie intake and weight loss [11,12]. The STEP clinical trial demonstrated that semaglutide produces significant weight loss in adults with obesity or overweight with comorbidities, leading to FDA approval for chronic weight management in these populations and showing better results than other GLP-1Ras [13].

Approved at a weekly dose of 2.4 mg for long-term weight management, semaglutide has shown better results than other GLP-1Ras [14,15]. In addition to reducing appetite, the drug may also help regulate fat tissue and boost energy expenditure. Still, its growing popularity among women of reproductive age raises important questions about safety during preconception and pregnancy. Animal studies have demonstrated potential risks to fetal development, including growth restrictions and skeletal abnormalities in rodent offspring exposed to semaglutide during gestation [16]. Human data remain limited; while a few observational studies of inadvertent first-trimester exposure have not identified a consistent increase in major congenital malformations, some have reported higher rates of preterm birth and large-for-gestational-age infants [17,18]. GLP-1 receptors are present in human placental cells, where they help regulate nutrient transfer and placental development [17].

In obese pregnancies, higher maternal GLP-1 levels have been linked to fetal growth, suggesting that administering exogenous GLP-1RAs could disrupt this balance [19]. While data on placental transfer of semaglutide remain limited, the growing off-label use of the drug for weight loss has led to more unintended pregnancies, highlighting the urgent need to understand its direct effects on placental function.

This study aims to explore the effects of semaglutide on two complementary types of human trophoblast cells: Swan71 cells, which model invasive extravillous trophoblasts responsible for uterine spiral artery remodeling, and BeWO cells, which exhibit syncytiotrophoblast characteristics and model the barrier and endocrine functions of the placenta. This dual-model approach allows evaluation of semaglutide effects across different trophoblast lineages. Therapeutic semaglutide dosing (2.4 mg weekly) produces steady-state serum concentrations of approximately 1–10 nM; however, tissue concentrations may be higher due to receptor binding and local accumulation. The goal is to determine whether semaglutide affects critical early pregnancy events including trophoblast proliferation, invasion, signaling pathway regulation, and metabolic function, ultimately providing mechanistic insight to guide clinical recommendations regarding semaglutide use during the periconception period.

## 2. Materials and Methods

### 2.1. Cell Culture and Treatments

Human BeWO cells, a choriocarcinoma-derived cell line with characteristics of normal syncytiotrophoblast phenotype (ATCC CCL-98, American Type Culture Collection, Manassas, VA, USA), and Swan71 cells, an immortalized first-trimester human trophoblast cell line (a generous gift from Dr. Gil Mor at Yale University [20]), were used in this study. Cells were cultured in DMEM/F12 medium (Gibco, Thermo Fisher Scientific, Waltham, MA, USA) for BeWO cells or RPMI 1640 medium (Gibco, Thermo Fisher Scientific, Waltham, MA, USA) for Swan71 cells, supplemented with 10% fetal bovine serum (FBS; Atlanta Biologicals, Flowery Branch, GA, USA) and 1% penicillin/streptomycin (Gibco, Thermo Fisher Scientific). At 80% cell confluency, trophoblast cells were treated with or without 100nM Semaglutide (Millipore Sigma, Burlington, MA, USA) for 24 h, and cell number and viability were determined. The 100 nM concentration was selected based on previous studies examining GLP-1RA effects in trophoblast and placental models and represents a concentration that may occur at the tissue level despite lower circulating concentrations [19,21], and it represents a concentration that may occur at the tissue level due to receptor binding and local accumulation, despite lower circulating steady-state plasma levels of approximately 1–10 nM for the 2.4 mg weekly dose. Following treatment, cells were harvested and counted using a hemocytometer. Cell viability was assessed by trypan blue (0.4%) exclusion which evaluates membrane integrity. Viable cells (excluding the dye) were counted and expressed as a percentage of total cells. BeWO cells, derived from choriocarcinoma, exhibit a malignant, glycolytic phenotype, whereas Swan71 cells represent immortalized, non-malignant first-trimester trophoblasts with a more oxidative metabolism.

Although therapeutic steady-state plasma concentrations of semaglutide are approximately 1–10 nM, the 100 nM concentration used here is consistent with prior in vitro studies examining GLP-1RA effects in trophoblast and placental models and is intended to reflect potential tissue-level exposure due to receptor binding and local accumulation [19,21].

### 2.2. Western Blot

Western blot analyses (n = 10) were conducted following procedures previously outlined by our laboratory [22]. Briefly, cells were lysed using RIPA buffer (Fisher Scientific, Saint Louis, MO, USA), and protein concentrations were determined using the bicinchoninic acid (BCA) protein assay kit (Thermo Fisher Scientific) according to the manufacturer’s instructions. Protein samples (30–35 µg) were separated on precast Mini-PROTEAN TGX gels (Bio-Rad Laboratories, Hercules, CA, USA) and transferred to nitrocellulose membranes. Membranes were incubated overnight at 4 °C with primary antibodies recognizing phosphorylated forms of mTOR (mechanistic target of rapamycin, a master regulator of cell growth and metabolism; Ser2448), p70 (70 kDa ribosomal protein S6 kinase, an mTOR downstream target regulating protein synthesis; Thr389), 4EBP1 (eukaryotic translation initiation factor 4E-binding protein 1, an mTOR target controlling translation initiation; Thr37/46), AKT (protein kinase B, a key mediator of growth factor signaling; Ser473), ERK (extracellular signal-regulated kinase, part of the MAPK pathway regulating proliferation and differentiation; Thr202/Tyr204) and AMPK (AMP-activated protein kinase, a cellular energy sensor Thr172), all from Cell Signaling Technology (Danvers, MA, USA). The next day, membranes were incubated with fluorophore-conjugated secondary antibodies for 60 min. Bands were visualized using a Li-COR Odyssey CLx imaging system. β-actin served as a loading control to correct for variations in protein input. Fluorescence band intensities were quantified using ImageJ software (version 1.54r/25 September 2025), and normalized values were used to compare experimental and control groups. Protein loading consistency was confirmed by pre-electrophoresis quantification and β-actin normalization. Densitometric values reflect the mean of independent biological replicates, performed under identical conditions to reduce technical variability.

### 2.3. Trophoblast Mitochondrial Respiration

To assess mitochondrial respiration, treated and control trophoblast cells were harvested, and oxygen consumption was measured at 37 °C using a high-resolution Oroboros O2k Oxygraph (Oroboros Instruments, Innsbruck, Austria) with MiR05 respiration buffer (Oroboros Instruments, Innsbruck, Austria). Basal respiration and electron flow through complex I were first evaluated in the presence of glutamate and malate (GM). To stimulate oxidative phosphorylation, adenosine diphosphate (ADP, 2.5 mM) was then added (GMD state). Subsequently, succinate was introduced (GMDS) to enable electron flow through both complex I and complex II to the Q-junction. Finally, the chemical uncoupler carbonyl cyanide 4-(trifluoromethoxy)phenylhydrazone (FCCP, 1µM) was added to determine maximal electron transport system (ETS) capacity beyond coupled oxidative phosphorylation. This sequential protocol allowed evaluation of mitochondrial respiratory efficiency and coupling under semaglutide treatment compared with controls.

### 2.4. Measurement of Reactive Oxygen Species (ROS)

Intracellular ROS production was assessed using the fluorogenic probe 2’,7’-dichlorodihydrofluorescein diacetate as suggested by the manufacturer (DCFH-DA; Sigma-Aldrich, St. Louis, MO, USA). Briefly, Swan71 and BeWO cells were plated at 1 × 10^4^ cells per well in black 96-well plates (Corning, New York, NY, USA) and allowed to attach overnight in their respective complete media (DMEM/F12 supplemented with 10% FBS for BeWO; RPMI with 10% FBS for Swan71). We prepared a fresh 10 μM working solution of DCFH-DA in HBSS from a 10 mM DMSO stock (stored at −20 °C in light-protected aliquots). Cells were incubated with 100 μL of 10 μM working solution of DCFH-DA per well for 45 min at 37 °C in the dark.

Following DCFH-DA incubation, cells were treated with 100 nM semaglutide or vehicle (PBS), cells were washed twice with PBS to clear any media remnants that might skew fluorescence. DCFH-DA pre-loading is a standard protocol for detecting subsequent intracellular ROS accumulation. We acknowledge that DCFH-DA is a general oxidative stress probe and may have some non-specificity toward different reactive species. Cells were kept in a light-free container and immediately transported for fluorescence measurement on a BioTek Synergy H1 microplate reader (excitation: 485 nm; emission: 535 nm; BioTeck Laboratories, Soreline, WA, USA) at 5-min intervals for 60 min, maintained at 37 °C. A positive control with 100 μM H2O2 (added at the time of treatment) was included on each plate to verify assay sensitivity, and a control with no cells, and semaglutide was also included. Separate vehicle controls were included for each cell line in its respective medium (DMEM/F12 for BeWo; RPMI for Swan71). Data are expressed as relative fluorescence units (RFU) after blank subtraction (HBSS-only wells) applied to all readings. Experiments were performed in triplicate wells across three independent runs (n = 9 total replicates).

### 2.5. Real-Time Cell Invasion

The invasive capacity of trophoblast cells, with or without Semaglutide treatment (n = 10), was evaluated using a real-time assay based on a protocol previously established in our laboratory [23]. Following the designated treatments, cell invasion was continuously monitored using the xCELLigence RTCA DP system (ACEA Biosciences, Blue Springs, MO, USA) and 16-well CIM-Plates. Prior to seeding, each well was coated with Matrigel (Fisher Scientific, Pittsburgh, PA, USA) at a 1:40 dilution to simulate the extracellular matrix. For each condition, 20,000 cells suspended in 100 µL of RPMI medium containing 2% FBS were placed in the upper chamber, while 160 µL of complete medium with 10% FBS was added to the lower chamber to establish a chemoattractant gradient. The instrument automatically recorded electrical impedance every 15 min for a 24-h period. Data were analyzed using the RTCA software (ACEA Biosciences, Blue Springs, MO, USA), which calculated invasion indices based on impedance changes corresponding to cell migration through the Matrigel barrier.

### 2.6. Statistical Analysis

All results are expressed as the mean ± standard error of the mean (SEM). Data distribution was evaluated to confirm normality before applying statistical tests. Comparisons between control and experimental groups were performed using the Mann–Whitney U test. A *p*-value less than 0.05 was considered statistically significant. Although data distribution was evaluated, the non-parametric Mann–Whitney U test was selected as a conservative approach given the sample sizes and inherent biological variability of the cell lines. Statistical analyses and graph generation were carried out using GraphPad Prism software, version 8.0 (GraphPad Software, San Diego, CA, USA).

## 3. Results

### 3.1. Semaglutide Impairs Trophoblast Cell Proliferation and Viability

**Figure 1:**
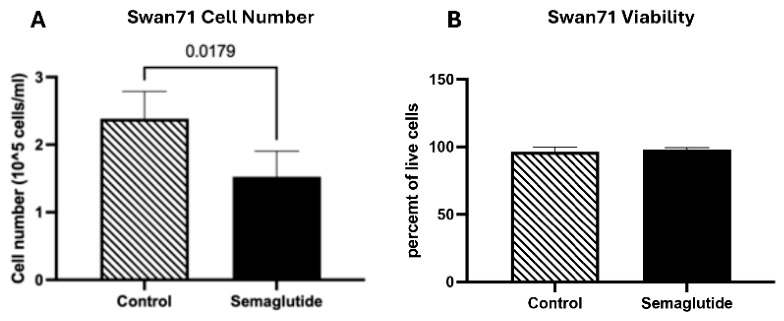
Effects of semaglutide on proliferation and viability in Swan71 trophoblast cells: (**A**) Cell counts (cells/mL) in control and semaglutide-treated cells. Data represent a 1.7-fold reduction (*p* = 0.0179) in cell number between control and treated cells. (**B**) No significant differences are observed in Cell viability (% live cells) between control and treated cells. Data are presented as mean ± SEM from independent experiments.

We first looked at how semaglutide affects cell numbers and survival in our trophoblast models. In the invasive Swan71 cells, semaglutide treatment significantly reduced cell counts from approximately 2.5 × 10^5^ cells/mL in controls to 1.5 × 10^5^ cells/mL (1.7-fold reduction; *p* = 0.0179; Figure 1A), while viability remained unchanged at approximately 100% in both groups (Figure 1B). these findings are consistent with reduced proliferation, as viability (membrane integrity) remained unchanged; however, we cannot fully exclude contributions from apoptosis without additional markers [19].

**Figure 2:**
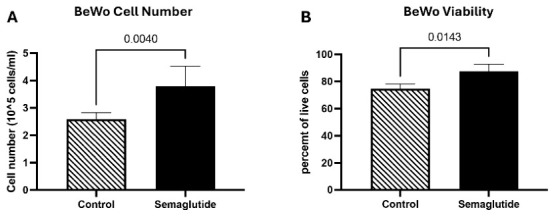
Effects of semaglutide on proliferation and viability in Bewo trophoblast cells: (**A**) Cell counts (cells/mL) in control and semaglutide-treated cells. Data represent a 1.8-fold increase (*p* < 0.0040) in cell numbers between control and treated cells. (**B**) Cell viability (% live cells) in control and treated cells; a slight increase from 75% to 88% (*p* = 0.0143) is observed in the treated cells as compared to controls. Data are presented as mean ± SEM from independent experiments. (**B**) No significant differences are observed in Cell viability (% live cells) between control and treated cells. Data are presented as mean ± SEM from independent experiments.

In the syncytiotrophoblast-like BeWO cells, a contrasting pattern was observed. Cell numbers increase from 2.6 × 10^5^ cells/mL in controls to 3.8 × 10^5^ cells/mL with semaglutide, a 1.8-fold increase as compared to controls (*p* < 0.0040; Figure 2A). Cell viability in control BeWO cells was 75% at 24 h, which increased slightly to 88% with semaglutide treatment (*p* = 0.0143; Figure 2B). Cells were initially plated at >95% viability, with the observed baseline reflecting typical BeWO cell characteristics after 24 h in culture. These differential responses between Swan71 and BeWO cells suggest cell-type-specific sensitivity to semaglutide.

Different mTOR Signaling Pathway Phosphorylation in Trophoblasts: To investigate the effects of semaglutide on trophoblast signaling pathways, Western blot analysis was performed to assess phosphorylated protein levels of key components in the mTOR pathway and related signaling cascades. Phosphorylated proteins were normalized to β-actin as a loading control.

**Figure 3:**
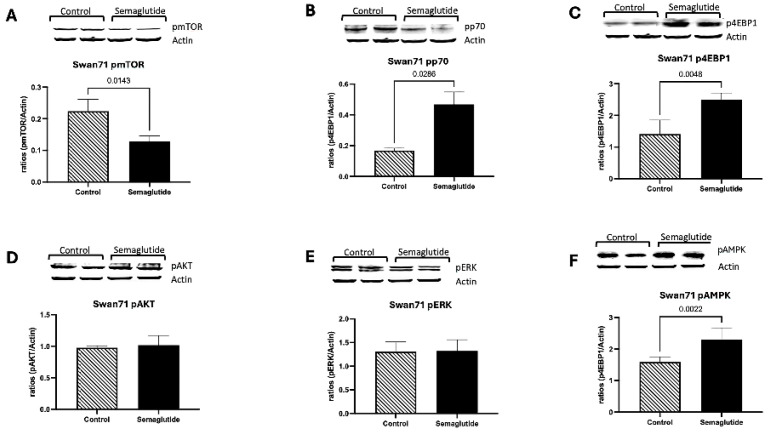
Effects of semaglutide on mTOR signaling pathway in Swan71 trophoblast cells. Western blot analysis (n = 10) of phosphorylated proteins is normalized to β-actin. A decrease in pmTOR (1.7-fold; *p* = 0.0040) is observed with semaglutide treatment as compared to controls (**A**). Semaglutide treatment increased both pp70S6K (2.7-fold; *p* = 0.0286) and p4EBP1 (1.9-fold; *p* = 0.0048) when compared to controls (**B**,**C**). No significant differences were observed in pAKT, and pERK, between semaglutide-treated and control groups (**D**,**E**). Trophoblast pAMPK was increased (1.9-fold increase, *p* = 0.0286) in semaglutide-treated cells as compared to controls. (**F**). Data are presented as mean ± SEM from independent biological replicates. ^a^ Independent biological replicates refer to experiments conducted on separate occasions using independently cultured and treated cells.

Swan71 Cells (Invasive Extravillous Trophoblast Model): In Swan71 cells, semaglutide treatment significantly decreased phosphorylated mTOR (pmTOR) levels by 1.7-fold compared to vehicle-treated controls (*p* = 0.0040; Figure 3A). In contrast, downstream targets of mTOR exhibited increased phosphorylation: pp70S6K levels increased 2.7-fold (*p* = 0.0286; Figure 3B), and p4EBP1 levels increased 1.9-fold (*p* = 0.0048; Figure 3C). This pattern of decreased pmTOR with increased phosphorylation of downstream targets is non-canonical and may reflect complex feedback regulation within the mTOR network or mTORC1-independent mechanisms. No significant changes were observed in phosphorylated AKT (pAKT) or phosphorylated ERK (pERK) levels between semaglutide-treated and control groups (Figure 3D,E). Notably, phosphorylated AMPK (pAMPK) levels increased 1.9-fold in semaglutide-treated cells compared to controls (*p* = 0.0286; Figure 3F), suggesting activation of this cellular energy-sensing kinase. These findings indicate complex alterations in mTOR pathway signaling with concurrent AMPK activation in Swan71 cells.

**Figure 4:**
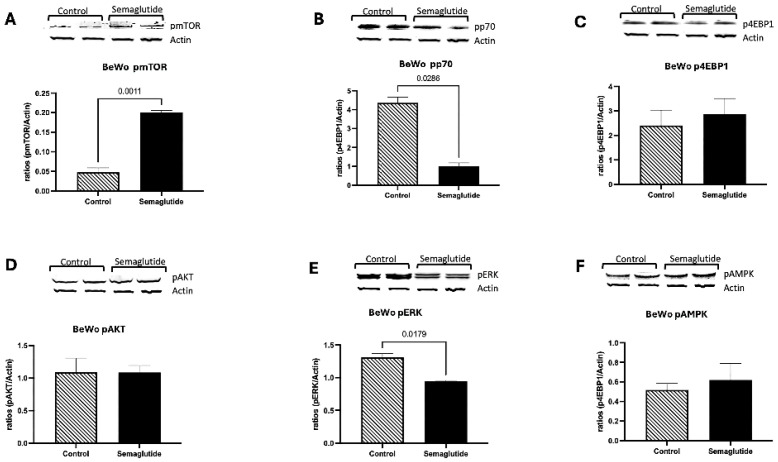
Effects of semaglutide on mTOR signaling pathway in BeWO trophoblast cells. Western blot analysis (n = 10) of phosphorylated proteins is normalized to β-actin. An increase in pmTOR (4.5-fold; *p* = 0.0143) is observed with semaglutide treatment as compared to controls (**A**). Trophoblast pp70S6K is decreased (4.4-fold; *p* = 0.0286) when cells are treated with Semaglutide (**B**). No significant differences are observed in p4EBP1 and pAKT, with semaglutide treatment compared to controls (**C**,**D**). pERK is significantly reduced (1.4-fold; *p* = 0.0317) while no changes in pAMPK are observed in the treated cells as compared to controls (**E**,**F**). Data are presented as mean ± SEM from independent biological replicates.

BeWO Cells (Syncytiotrophoblast Model): In BeWO cells, the mTOR signaling response to semaglutide differed substantially from that observed in Swan71 cells. Phosphorylated mTOR (pmTOR) levels increased 4.5-fold in semaglutide-treated cells compared to controls (*p* = 0.0143; Figure 4A). Conversely, the downstream target pp70S6K exhibited a significant decrease of 4.4-fold with semaglutide treatment (*p* = 0.0286; Figure 4B). Phosphorylated 4EBP1 (p4EBP1) and phosphorylated AKT (pAKT) levels remained unchanged following semaglutide treatment (Figure 4C,D).

Phosphorylated ERK (pERK) levels decreased significantly by 1.4-fold in treated cells compared to controls (*p* = 0.0317; Figure 4E), while no significant changes were observed in pAMPK levels (Figure 4F).

These results demonstrate that semaglutide exerts divergent, lineage-specific effects on mTOR pathway signaling in trophoblast cells, with opposing patterns of mTOR and downstream effector phosphorylation between invasive Swan71 and syncytial BeWO cell models.

### 3.2. Semaglutide Alters Mitochondrial Oxygen Consumption

**Figure 5:**
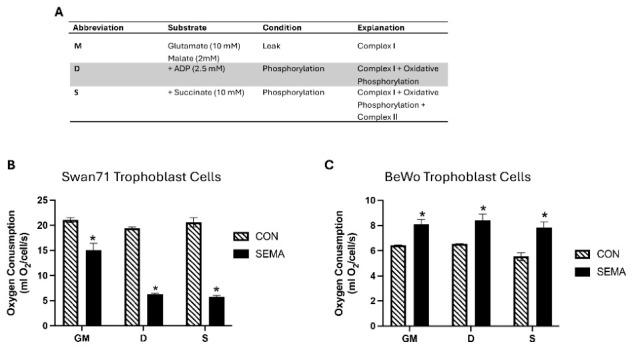
Mitochondrial respiration in semaglutide-treated trophoblast cells. Mitochondrial respiration components are shown in (**A**). Treated Swan71 cells show a general decrease (*p* < 0.05) in mitochondrial respiration when compared to controls (**B**). In contrast, BeWO trophoblast showed a general increase (*p* < 0.05) in mitochondrial function when treated with semaglutide (**C**). Data are presented as mean ± SEM from independent experiments. * *p* < 0.05 vs. control.

To examine mitochondrial function, oxygen consumption was measured under sequential substrate conditions (Figure 5A). Semaglutide significantly reduced mitochondrial respiration in Swan71 cells under glutamate, malate, and succinate-supported conditions (*p* < 0.05; Figure 5B). In contrast, BeWO cells exhibited a significant increase in oxygen consumption compared with controls (*p* < 0.05; Figure 5C). These contrasting metabolic responses further highlight distinct energy regulation mechanisms between invasive and syncytial trophoblast lineages.

### 3.3. Semaglutide Elevates Reactive Oxygen Species in Trophoblast Cells

**Figure 6:**
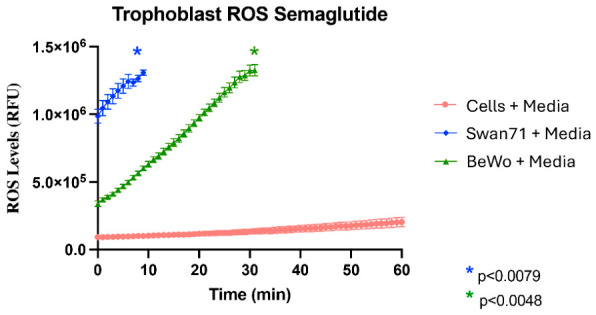
Semaglutide-induced reactive oxygen species (ROS) production in trophoblast cells. Time course of intracellular ROS levels measured by DCFH-DA fluorescence in Swan71 and BeWO cells treated with semaglutide (100 nM) or vehicle control. Measurements are performed continuously for 60 min at 5-min intervals; the displayed time range (0–15 min for Swan71, 0–40 min for BeWO) focuses on the period of dynamic ROS accumulation, as ROS levels plateau after reaching peak values. Swan71 cells (blue) show a 13-fold increase in ROS levels (relative fluorescence units, RFU) peaking at 9 min (*p* < 0.0079); BeWO cells (green) show a 13.2-fold increase peaking at 31 min (*p* < 0.0048). Vehicle-treated control cells (red) maintain low, stable ROS levels throughout. Statistical significance is determined by Mann–Whitney U test comparing peak values to respective controls. Data represent mean ± SEM from three independent experiments with triplicate wells (n = 9 total).

Oxidative stress was evaluated by measuring intracellular reactive oxygen species (ROS) production using DCFH-DA fluorescence. Fluorescence was monitored continuously for 60 min following semaglutide or vehicle treatment (Figure 6). Semaglutide significantly elevated ROS levels in both cell lines compared to controls. In Swan71 cells, ROS levels increased from baseline to peak at 1.4 × 10^6^ RFU by 9 min (13-fold increase; *p* < 0.0079). BeWO cells exhibited a similar pattern with ROS rising to 1.3 × 10^6^ RFU by 31 min (13.2-fold increase; *p* < 0.0048). Control cells maintained relatively stable ROS levels around 0.2–0.3 × 10^6^ RFU throughout the measurement period.

### 3.4. Semaglutide Hinders Trophoblast Invasion

**Figure 7:**
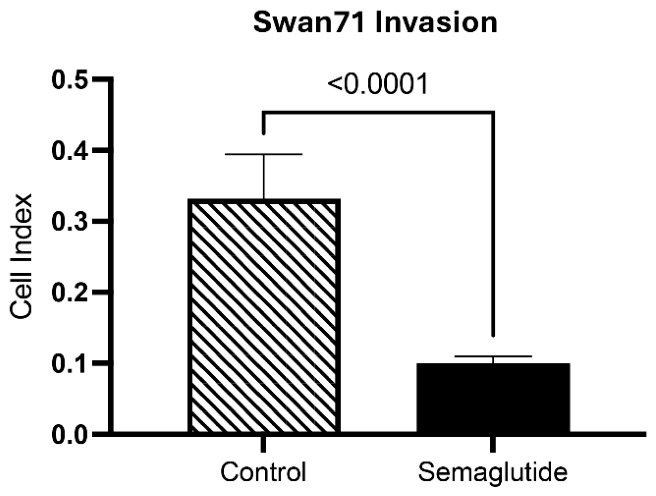
Effects of semaglutide on trophoblast cell invasion. Real-time invasion of Swan71 cells (n = 10 independent experiments) through Matrigel-coated membranes is monitored using the xCELLigence RTCA DP system. The y-axis represents the cell index; a dimensionless parameter derived from electrical impedance measurements that reflects the number of cells attached to the membrane. Higher cell index values indicate greater invasion. Semaglutide treatment (100 nM, 24 h) significantly decreases invasion by 3.0-fold (*p* < 0.0001) compared to vehicle-treated controls. Data are presented as mean ± SEM from 10 independent biological replicates.

Trophoblast invasiveness was assessed using a Matrigel invasion assay only in Swan71 cells, as this cell line models the invasive extravillous trophoblast phenotype responsible for uterine spiral artery remodeling during placentation. BeWO cells, which model the non-invasive syncytiotrophoblast layer, were not evaluated for invasion. Semaglutide significantly reduced the invasion index from 0.3 in controls to 0.1—a 3.0-fold drop (*p* < 0.0001; Figure 7). The invasion index, derived from impedance measurements, reflects the number of cells that successfully migrated through the Matrigel barrier, with lower values indicating impaired invasive capacity. This pronounced inhibition suggests that semaglutide may reduce invasive capacity, which could contribute to impaired placental remodeling [24].

## 4. Discussion

Our findings demonstrate that semaglutide exerts distinct and multifaceted effects on human trophoblast cell lines, revealing potential consequences for placental function during pregnancy. In invasive Swan71 cells, which model extravillous trophoblasts, semaglutide markedly reduced cell numbers, although cell viability remained unchanged. In contrast, BeWO cells—representing syncytiotrophoblasts—displayed increased proliferation and a modest rise in viability. These opposite responses indicate that semaglutide influences trophoblast growth in a lineage-specific manner: it appears to suppress proliferation in invasive trophoblasts while stimulating expansion in syncytial-like cells. These divergent proliferation responses contrast with findings from other GLP-1RA studies in placental models. For instance, Qiao et al. (2024) [19] reported that maternal GLP-1 receptor activation in pregnant mice resulted in reduced fetal and placental weight, suggesting an overall growth-inhibitory effect. However, their study examined systemic GLP-1 receptor activation during pregnancy, whereas our findings reveal that direct effects on isolated trophoblast populations may be cell-type dependent. Similarly, Dumolt et al. (2023) [24] found that endogenous maternal GLP-1 levels were positively associated with fetal growth in obese pregnancies, highlighting the complexity of GLP-1 signaling in the placental-fetal unit. These discrepancies underscore the need to distinguish between physiological GLP-1 receptor signaling and pharmacological perturbation with long-acting agonists like semaglutide.

At the signaling level, semaglutide substantially modified several components of the mTOR pathway, a key regulator of trophoblast growth and metabolism. In Swan71 cells, phosphorylation of mTOR and AKT decreased, while phosphorylation of p70S6K and 4EBP1 increased, and AMPK activation was also elevated. This pattern suggests a complex regulatory shift characterized by mTOR inhibition with partial activation of its downstream effectors, consistent with a metabolic transition toward a more catabolic state. In BeWO cells, semaglutide increased p-mTOR but reduced p-p70S6K and ERK phosphorylation, reflecting dysregulation rather than uniform suppression of these pathways. Because ERK activity supports trophoblast proliferation and syncytialization, its reduction implies that semaglutide could impair differentiation as well as growth. The disruption of mTOR-AMPK signaling observed here parallels findings in other cell types exposed to GLP-1RAs. In pancreatic beta cells and hepatocytes, GLP-1 receptor activation has been shown to modulate mTOR activity and promote AMPK phosphorylation as part of metabolic adaptation [25]. However, the consequence of such signaling changes in trophoblasts—cells critically dependent on mTOR for nutrient sensing and growth—may be particularly detrimental during early placental development. These findings align with semaglutide’s known systemic actions on nutrient sensing and energy balance and may contribute to impaired placental growth and invasion [12].

Semaglutide also heightened oxidative stress in both trophoblast models. ROS levels rose rapidly after treatment and remained significantly elevated compared with controls. In Swan71 cells, mitochondrial respiration decreased under substrate-supported conditions, while BeWO cells exhibited an overall increase, suggesting lineage-specific metabolic adaptations. These results parallel animal studies showing that semaglutide alters placental lipid metabolism and redox homeostasis [19]. Oxidative stress is a defining feature of placental disorders such as preeclampsia and fetal growth restriction [26], raising concern that semaglutide exposure may disrupt the redox balance required for normal placental development. Although GLP-1RA agonists can reduce oxidative stress in other reproductive tissues [24], our findings indicate that trophoblasts respond differently, possibly through mechanisms secondary to mTOR-AMPK pathway alteration. The observed ERK suppression in BeWO cells further supports this, as MAPK/ERK signaling contributes to proliferation and syncytial fusion. Reduced ERK activity could therefore compromise trophoblast differentiation and hormone production, providing a mechanistic link between semaglutide exposure and impaired placental adaptation. The elevation of oxidative stress observed in both trophoblast models warrants comparison with effects of GLP-1RA in other reproductive contexts. Zhang et al. (2025) [27] recently reported that semaglutide treatment in obese female mice prior to pregnancy ameliorated oxidative stress in oocytes and improved metabolic parameters in offspring, suggesting beneficial effects when administered before conception. This contrasts sharply with our findings of increased ROS in trophoblast cells during treatment. This discrepancy may reflect critical differences between preconception metabolic optimization versus exposure during active placental development, when trophoblasts are particularly vulnerable to oxidative damage [26]. Furthermore, while GLP-1RA have demonstrated antioxidant properties in some tissue contexts—such as cardiovascular and neural tissues [28]—our data indicate that trophoblasts may respond differently, possibly due to their unique metabolic demands and reliance on tightly regulated redox balance for proper differentiation and invasion. Oxidative stress is a well-established feature of placental pathologies including preeclampsia and intrauterine growth restriction [26], raising concern that semaglutide-induced ROS generation could contribute to placental dysfunction if exposure occurs during critical developmental windows.

Consistent with these molecular changes, semaglutide markedly decreased the invasive capacity of Swan71 cells. Trophoblast invasion is essential for proper anchoring of the placenta and remodeling of maternal spiral arteries, and its inhibition may predispose to shallow placentation and reduced uteroplacental perfusion [16]. Collectively, these findings indicate that semaglutide induces a stress-like phenotype in trophoblasts—characterized by altered signaling, increased oxidative stress, disrupted mitochondrial function, and reduced invasion. Together, these cellular responses could underlie placental dysfunction associated with GLP-1RA exposure during early pregnancy.

The marked suppression of trophoblast invasion observed in Swan71 cells is consistent with animal studies demonstrating impaired placental vascular development following GLP-1RA exposure. Wang et al. (2025) [29] reported reduced placental vascularization and altered fetal hypothalamic development in rat offspring when semaglutide was administered prior to pregnancy, though effects were sex-specific. Similarly, preclinical studies have linked GLP-1 receptor activation to restricted placental growth and compromised uteroplacental blood flow [16,19]. Adequate trophoblast invasion is essential for spiral artery remodeling and establishment of maternal-fetal circulation; thus, the three-fold reduction in invasion we observed could have implications for placental perfusion and nutrient delivery if it occurs in vivo. Several limitations should be acknowledged. This study utilized established trophoblast cell lines rather than primary placental tissue, applied a single semaglutide concentration (100 nM), which exceeds typical circulating therapeutic plasma levels (~1–10 nM), and examined only a 24-h exposure period. Although the concentration was chosen based on prior trophoblast studies and may approximate tissue-level exposure due to receptor binding and local accumulation, it remains higher than steady-state plasma concentrations. While GLP-1 receptors have been reported in human placental tissue, we did not confirm GLP-1R expression in the Swan71 and BeWO cell lines used in this study; therefore, the observed effects may involve both receptor-mediated and off-target mechanisms. The proliferation and viability data are based solely on cell counting and trypan blue exclusion and do not distinguish between reduced proliferation, cell cycle arrest, or apoptosis. Similarly, although ROS levels were elevated, the study is correlative and does not establish causality between oxidative stress and the observed phenotypes (e.g., impaired invasion or altered signaling). The mitochondrial respiration data would also benefit from additional normalization parameters (e.g., coupling efficiency) in future studies. These limitations notwithstanding, the present findings provide mechanistic insight into potential direct effects of semaglutide on trophoblast function and warrant caution regarding its use during the periconception period.

## 5. Conclusions

In summary, semaglutide altered several key aspects of trophoblast biology in vitro. Treatment with semaglutide reduced proliferation and invasion in Swan71 trophoblasts, increased proliferation in BeWO syncytial cells, and disrupted normal signaling through the mTOR and MAPK/ERK pathways. These molecular effects were accompanied by increased AMPK activation, elevated reactive oxygen species, and changes in mitochondrial respiration, collectively suggesting heightened metabolic and oxidative stress. Together, these findings suggest that semaglutide may alter the adaptive responses of trophoblasts that are required for proper placental remodeling and nutrient exchange. Given the rapid rise in GLP-1RA use among women of reproductive age, understanding their potential impact on early pregnancy is increasingly important. While preconception treatment with semaglutide may improve metabolic health and reduce obesity-related risks, continued exposure around the time of conception could interfere with implantation and placentation. Current recommendations advising discontinuation of GLP-1RA at least two months before attempting conception remain appropriate [26]. Future studies should explore dose-dependent and time-dependent effects, verify GLP-1 receptor expression, and distinguish receptor-mediated from off-target mechanisms. As new incretin-based therapies become more widely available, careful investigation of their reproductive safety will be essential. The present study provides mechanistic insight into how semaglutide influences trophoblast signaling and metabolism and underscores the importance of assessing placental health alongside metabolic efficacy in future clinical and preclinical research.

## Data Availability

The original contributions presented in the study are included in the article.

## Abbreviations

The following abbreviations are used in this manuscript:

AKT	Protein kinase B
AMPK	AMP-activated protein kinase
BMI	Body mass index
DCFH-DA	2′,7′-Dichlorodihydrofluorescein diacetate
ERK	Extracellular signal-regulated kinase
GDM	Gestational diabetes mellitus
GLP-1	Glucagon-like peptide-1
GLP-1RA	Glucagon-like peptide-1 receptor agonist
MAPK	Mitogen-activated protein kinase
RFU	Relative fluorescence units
ROS	Reactive oxygen species
SEM	Standard error of the mean
WHO	World Health Organization
mTOR	Mechanistic target of rapamycin
p4EBP1	Phosphorylated eukaryotic translation initiation factor 4E-binding protein 1
p70S6K	70 kDa ribosomal protein S6 kinase
pAKT	Phosphorylated protein kinase B
pAMPK	Phosphorylated AMP-activated protein kinase
pERK	Phosphorylated extracellular signal-regulated kinase
pmTOR	Phosphorylated mechanistic target of rapamycin
pp70S6K	Phosphorylated 70 kDa ribosomal protein S6 kinase
